# Water metagenomes reflect physicochemical water quality throughout a model agricultural pond

**DOI:** 10.3389/fmicb.2025.1535096

**Published:** 2025-06-04

**Authors:** Ryan A. Blaustein, Jaclyn E. Smith, Magaly Toro, Yakov Pachepsky, Matthew D. Stocker

**Affiliations:** ^1^Department of Nutrition and Food Science, University of Maryland, College Park, MD, United States; ^2^Oak Ridge Institutes for Science and Education, Oak Ridge, TN, United States; ^3^United States Department of Agriculture, Agricultural Research Service, Environmental Microbial and Food Safety Laboratory, Beltsville, MD, United States; ^4^Joint Institute for Food Safety and Applied Nutrition, University of Maryland, College Park, MD, United States

**Keywords:** water microbiome, agriculture, water quality, metagenomics, antimicrobial resistance

## Abstract

Agricultural ponds are essential irrigation resources, though may also serve as reservoirs for pathogens and antimicrobial resistance (AMR) genes. While monitoring microbiological water quality is critical for food safety, the influence of sampling factors (e.g., when and where to collect samples) in making risk assessments and potential applications for using environmental covariates as indicators remain unclear. Here, we explored the hypothesis that metagenomes of agricultural waters change with spatiotemporal shifts in physicochemical water quality, i.e., across water depths over time. Water samples and underlying sediments were collected at a model pond at the surface and within the water column (0, 1, 2 m depths) throughout one day (i.e., 9:00, 12:00, 15:00). All samples were processed for shotgun metagenomic sequencing analysis and enumeration of various water quality parameters (e.g., temperature, nutrient concentrations, turbidity, pH, culturable *Escherichia coli*). At the pond surface, *Microcystis aeruginosa* and members of Cyanobacteria, along with genes encoding pathways related to photosynthesis and nucleotide biosynthesis, were enriched throughout the day. In contrast, within the water column (1–2 m depths) and sediments, diverse members of Proteobacteria and Actinobacteria were more dominant, along with encoded pathways related to respiration and amino acid biosynthesis. Various aspects of water quality (i.e., chlorophyll dissolved organic matter, ammonia, *E. coli* concentrations) correlated with water metagenome diversity, albeit not with any specific AMR genes or virulence factors. Nevertheless, *de novo* assembly of sequenced reads uncovered 22 unique strains encoding several AMR, virulence, or stress response genetic elements, thus linking metagenome functional potential to key taxa. Overall, our findings highlight distinctions in agricultural pond water metagenomes at the surface and in the water column and demonstrate the potential for metagenomic surveillance in water quality monitoring to support food safety.

## 1 Introduction

Aquatic microbiomes contain diverse planktonic and sediment-resuspended bacteria, viruses, algae, and fungi that collectively play important roles in nutrient biotransformation and cycling, decomposition of organic matter, and transmission and degradation of biological and chemical contaminants, among other ecosystem functions (Andreote et al., 2018; Escalas et al., 2019; Rout et al., 2024a). Abiotic factors (e.g., temperature, nutrient availability, salinity) vary spatially and temporally in surface waters, including gradients along different depths or flow channels (Neneng et al., 2020). The water quality properties and the intrinsic microbiota both fluctuate at broad and fine spatial and temporal scales, such as between seasons or even within a given day (Newton et al., 2011; Mayr et al., 2020; Stocker et al., 2024; Zhao et al., 2024). Changes in temperature, dissolved oxygen, and nutrient influx in salt marshes have been attributed to restructuring of the aquatic microbial community (Kearns et al., 2016). Likewise, populations of cyanobacteria among the broader water microbiome have been reported to respond to diurnal changes in UV light intensity at different depths within lakes (Cameron et al., 2024). As such, key members of water microbiomes have been suggested as novel indicators for predicting pollution and other aspects of water quality (Wang et al., 2020; Stocker et al., 2024).

To date, most research on water microbiomes has applied 16S rRNA gene sequencing for taxonomic characterization or used more traditional culture-dependent approaches (Andreote et al., 2018; Wang et al., 2020; Cameron et al., 2024; Stocker et al., 2024; Zhao et al., 2024). Understanding relationships between water quality and microbiome functional potential remains largely unexplored. Shotgun metagenomic sequencing is a powerful tool that is increasingly used for microbiome taxonomic and functional profiling. Using deep metagenomic sequencing, He et al. (2024) discovered novel lineages of SAR202 bacteria and their encoded functions, which play important roles in global carbon cycling at different ocean depths. Another recent report on metagenomes in surface water samples in Chile and Brazil (*n* = 404 metagenomes) identified a variety of microbial contaminants (i.e., *Escherichia*, *Listeria*, *Salmonella*) and diverse antimicrobial resistance (AMR) genes spanning 25 antimicrobial classes (Huang et al., 2024). Advancements in molecular surveillance tools to monitor changes in water quality and for genetic elements involved in AMR, virulence, or even microbial production of toxins (e.g., cyanotoxins) in water resources warrant further investigation for applications to support public health (Escalas et al., 2019; Liguori et al., 2022; Linz et al., 2023).

The microbial quality of agricultural irrigation water is a critical factor for food safety. The U.S. Food and Drug Administration (FDA) recently released the final rule on pre-harvest water as part of the Food Safety Modernization Act Produce Safety rule, requiring farms to perform thorough water quality evaluations, as least on an annual basis, for risk management decision-making purposes (U.S. Food and Drug Administration, 2024). Nevertheless, the influence of water sampling factors (e.g., numbers and volume of samples, water depth, timing of collection) and analytical frameworks on predictive outcomes is not well understood. Moreover, agricultural ponds are an important component in the “one-health” narrative as a sink and source of foodborne pathogens and AMR genes from the environment to fresh produce for human consumption (Franklin et al., 2024). While monitoring pond metagenomes has demonstrated seasonal or regional variations in microbial diversity, AMR, and functional profiles (Chopyk et al., 2020; Malayil et al., 2022), understanding the relationships between microbiome functional potential and environmental covariates associated with water quality (e.g., nutrient concentrations, turbidity) hold potential as novel indicators and improving monitoring recommendations.

Our previous work that utilized 16S rRNA gene sequencing demonstrated that bacterial community taxonomic diversity exhibits variation throughout a model pond at different depths over the course of the day (i.e., 9:00, 12:00, 15:00), with implications for improving water quality monitoring strategies (Stocker et al., 2024). Expanding upon these findings, the present study aimed to explore fine-scale spatial and temporal variations in the pond metagenomes to (1) determine differences at the surface and at depths in the water column over time to establish the impacts of sampling design on the observed microbiome diversity and (2) identify associations between metagenome functional potential (i.e., encoded pathways, AMR genes, virulence factors) and an array of water quality properties.

## 2 Methods

### 2.1 Sample collection, DNA extraction, and sequencing

Sampling was conducted at an agricultural pond at the University of Maryland’s Wye Research and Education Center on 09/15/2022, as reported previously (Stocker et al., 2024). The pond that was sampled (38.916 N 76.141 W) had an approximate surface area of 4,000 m^2^ with average depth of 3 m. Here, we extended the original study by processing a subset of the water and sediment samples, which were collected at one site nearshore and two sites at the interior transect of the pond (i.e., L4, L5, and L11 in Stocker et al., 2024; Supplementary Figure S1), for shotgun metagenomics analysis. The sample selection enabled investigation of differences in pond water and sediment metagenomes as a factor of sampling location (*n* = 3 within-pond sites) and water metagenomes further as a factor of sampling depth (*n* = 3 gradient depths at L5 and L11) and time (*n* = 3 time points at all locations). Specifically, surface water samples were collected at all locations from boat at 9:00, 12:00, and 15:00 using sterile 500 mL bottles with aseptic technique (*n* = 9 water samples taken at the surface, i.e., 3 locations × 3 times). Gradient depth samples in the water column were also collected at these times at L5 and L11 at 1 m and 2 m depths (*n* = 12 water samples taken at depth, i.e., 2 locations × 2 depths × 3 times). We used a weighted strainer attached to depth-marked vinyl tubing connected to a peristaltic pump to fill the bottles (SigmaMAX 900, Loveland, CO, United States) following an initial 30 s flush. Sediment cores were then collected after the final timepoint at 15:00 at each location (*n* = 3 sediment samples taken, one at each location), i.e., to avoid microbial resuspensions during the water sampling events. The sediment was sampled at the bank site (L4) using sterile 50 mL conical tubes and at the internal sites (L5, L11) using an Eckman dredge.

DNA was extracted from the water and sediment samples using the DNeasy PowerWater Kit (Qiagen, Hilden, Germany) (Stocker et al., 2024). Water samples (50 mL) and mixed sediment samples (i.e., slurries prepared from 3 g sediment vortexed in 27 mL sterile DI water; 1:10 dilution) were passed through 0.45 μm filters using a manifold vacuum. DNA was extracted from the filters and quantified with a Qubit 4 Fluorometer (Invitrogen, Waltham, MA) (*n* = 24 samples, 20.0 ± 2.4 ng/μl, mean ± standard error). Negative controls included DNA extractions from DI water passed through the same filters (*n* = 1) and reagent blanks (*n* = 3) that were processed alongside water/sediment samples, all of which yielded DNA below detection range. Metagenome libraries were prepared with the Illumina DNA Prep kit with UD Indexes (Illumina, San Diego, CA). Paired-end sequencing (300 cycles) targeting >15 million reads per sample was performed on an Illumina NextSeq1000 at the Joint Institute for Food Safety and Applied Nutrition.

### 2.2 Metagenome analysis

Raw sequenced reads were processed to remove potential sequencing contaminants and low-quality reads using Kneaddata v.0.6.1 with default parameters.^1^ Microbiome taxonomic classification was performed with Kraken2 v2.1.2 (Wood et al., 2019) with the option “–confidence 0.1” (Saheb Kashaf et al., 2022; Blaustein et al., 2023), followed by species-level read correction with Braken (Lu et al., 2017). Metagenome functional profiling was performed using HUMAnN2 v.0.11.1 (Franzosa et al., 2018) with default parameters, and MetaCyc pathway abundances were approximated based on copies per million (CPM) of mapped reads. AMR genetic element and virulence factor (VF) reads per kilobase per million reads (RPKMs) were determined with ShortBRED v0.9.5 (Kaminski et al., 2015) by running *shortbred_quantify* with *shortbred_identify* markers (length >15 amino acids) constructed from protein sequences of the Comprehensive Antibiotic Resistance Database (CARD) v3.2.7 (Alcock et al., 2020) (accessed 08/11/2023) and the Virulence Factor Database (Liu et al., 2022) (VFDB; accessed 08/27/2023), each with reference to UniRef50 (Suzek et al., 2015).

We further employed *de novo* assembly to recover metagenome-assembled genomes (MAGs) as previously described (Blaustein et al., 2023). In brief, metaSPAdes v.3.15.0 (Bankevich et al., 2012), MetaWRAP v.1.2.2 (Uritskiy et al., 2018), and GUNC v1.0.5 (Orakov et al., 2021) were used, each with default parameters, for assembly, binning (with concoct, maxbin2, and metabat2), and prediction and removal of chimeric MAGs, respectively. For the latter, MAGs with contamination greater than 0.05, clade separation greater than 0.45 and a reference representation score greater than 0.5 were considered chimeric and removed (Saheb Kashaf et al., 2022). We then used dRep v2.6.2 (Olm et al., 2017) to dereplicate refined bins with predicted completeness greater than 50% and contamination less than 5% based on an average nucleotide identity (ANI) of 99% and minimum overlap of 30% (i.e., to approximate the strain level). The same parameters with 95% ANI were used to refine dereplicated MAGs to the species level. MAGs were given taxonomic assignment using GTDB-Tk v1.3.0 (Chaumeil et al., 2022) and novelty was established based on relative evolutionary divergence (RED) score. The generated protein sequence alignments were used to construct a phylogenetic tree via FastTree v.2.1.10 (Price et al., 2010) that was visualized with ape (Paradis and Schliep, 2019). MAGs were then queried for genes encoding proteins with homology to AMR and virulence-associated features using AMRFinderPlus v3.12.8 (Feldgarden et al., 2021) (database 07-22-2024) with settings for 50% amino acid alignment identity (i.e., since the MAGs are non-model organisms) and the flag “--plus” to return the comprehensive set of AMR, virulence, and stress response genes.

### 2.3 Water quality

Water quality properties associated with each sample were measured *in situ* and via laboratory analysis. In the field, a water sample collection strainer was attached to the sensor guard of a YSI EXO-2 multiparameter water quality sonde that was calibrated with manufacturer standards prior to field use (Yellow Springs Instruments, Yellow Springs, Ohio, United States). The sonde provided *in situ* measurements of temperature (°C), dissolved oxygen (DO) (mg L^−1^), specific conductivity (SPC) (μS cm^−1^), turbidity (NTU), phycocyanin (YSI-PC) (relative fluorescent unit; RFU), chlorophyll-a (YSI-CHL) (RFU), and fluorescent dissolved organic matter (FDOM) (μg L^−1^). At the lab, aliquots of the water samples were processed for *Escherichia coli* concentrations (CFU 100 mL^−1^) via enumeration using membrane filtration and modified mTEC agar (ECCFU) (BD Difco, Sparks, Maryland). The water samples were also processed to measure phycocyanin (PHYC) (μg L^−1^), chlorophyll-a (INV) (RFU), colored dissolved organic matter (CDOM) (μg L^−1^), and nutrient concentrations of orthophosphate (PO4) (mg L^−1^), nitrate (NO3) (mg L^−1^), dissolved ammonia (NH3) (mg L^−1^), total organic carbon (TOC) (mg L^−1^), total inorganic carbon (TIC) (mg L^−1^), and total nitrogen (TN) (mg L^−1^) using methods described in our previous study (Stocker et al., 2024).

### 2.4 Statistical analysis

All statistics and data visualizations were completed in R v.4.3.1. Vegan^2^ was used to compute α -diversity (Shannon index) and β -diversity (Bray–Curtis dissimilarity) for microbiome taxonomic profiles as relative abundances at the species level and functional profiles as relative abundances of MetaCyc pathway CPMs. The effects of sampling factors (i.e., sampling location, water column depth, time of day) on microbiome α -diversity and β -diversity were determined with ANOVA and PERMANOVA, respectively. Correlations with the measured water quality properties were evaluated with the Spearman’s rank test ( α -diversity) and PERMANOVA ( β -diversity) as well. A linear mixed-effects (LME) model was employed to determine differential abundances of microbial species (i.e., those that were >0.1% abundant on averages across all samples), MetaCyc pathways, and AMR and VF genetic elements based on fixed effects for the sampling depth (i.e., water surface, water column, sediments) and random effects for sampling location (i.e., L4, L5, L11) and time of day (i.e., 9:00, 12:00, 15:00). *p*-values were adjusted with Benjamini-Hochberg correction and *q* < 0.05 was considered significant. The total number of genes per MAG with homology to AMR and VF genetic elements was compared for MAGs belonging to different bacterial phyla using the Dunn’s test with Benjamini-Hochberg correction.

## 3 Results

### 3.1 Pond water and sediment metagenomic profiles correlate with sampling factors and water quality

Metagenomic sequencing of the water (*n* = 21) and sediment (*n* = 3) samples generated 302.8 M sequenced reads (or 200 Gb data), with 12.6 ± 2.6 M paired end reads per sample (mean ± standard deviation). After pre-processing, there were 8.8 ± 2.0 M high quality non-human paired reads per sample from which 1,303 microbial species representing 613 genera, 238 families, 134 orders, 70 classes, and 33 phyla were detected.

**Figure 1:**
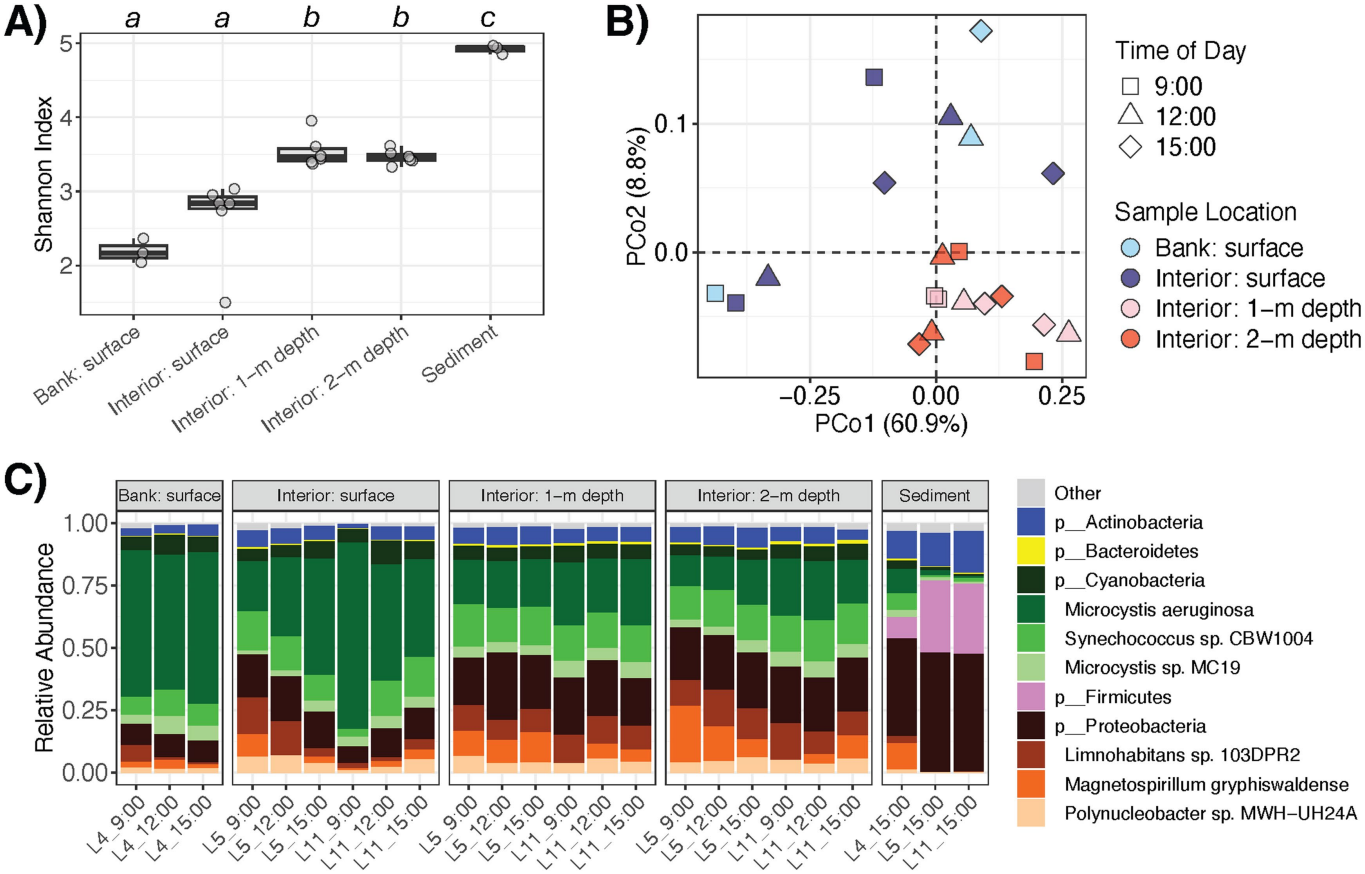
Composition and diversity of pond microbiomes at different sampling locations or cross-sectional depths of the pond. **(A)** α-diversity of the water and sediment microbiomes (Shannon index). Letters indicate significant difference (*p* < 0.05) based on ANOVA and Tukey’s *post-hoc* test. **(B)** Compositional dissimilarity or β-diversity of the water microbiomes. Colors and shapes correspond to sampling location and time of day, respectively. **(C)** Relative abundances of microbial taxa in the water and sediment microbiomes. Color corresponds to phylum or genus.

Pond microbiome α -diversity was lowest at the water surface, especially near shore (L4) compared to the interior sites (L5 and L11), and it was significantly higher within the water column and highest in the pond sediments (Figure 1A). At L5 and L11, the water microbiomes at the surface exhibited significantly lower α -diversity as compared to those at the 1 m (*q* = 0.002) and 2 m (*q* = 0.004) depths throughout the day, though there were no differences detected between the 1 m and 2 m depths (*q =* 0.977). Accordingly, microbial community profiles at the water surface (all sites) and those in the water column (i.e., L5 and L11 at depths of 1 m and 2 m) clustered apart (PERMANOVA *R*^2^ = 0.206, *p* = 0.008) (Figure 1B). On the contrary, the time of day (i.e., 9:00, 12:00, 15:00) did not appear to significantly associate with α -diversity (*p* = 0.669) or β -diversity (PERMANOVA *R*^2^ = 0.132, *p* = 0.228) of the water microbiota. Thus, the pond water microbiomes were substantially different between the surface and depths in the water column with variation that appeared to be consistent throughout the day.

**Figure 2:**
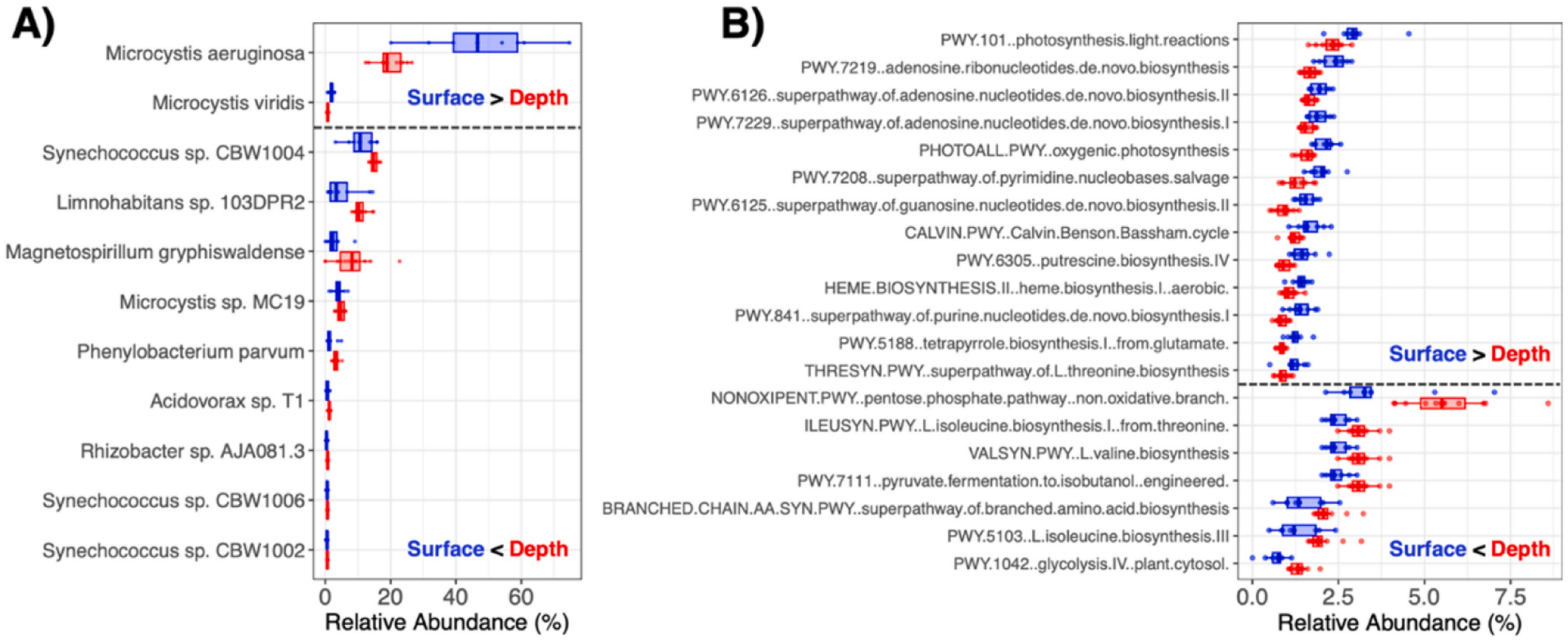
Microbiome taxa and functional features that were enriched at the pond water surface vs. within the water column. **(A)** Microbial species with average relative abundance >0.5% that were significantly different (*q* < 0.05) between the pond water surface and within the depths of the water column. **(B)** MetaCyc pathways with average relative abundance >1% that were significantly different (*q* < 0.05) between the pond water surface and within the depths of the water column.

The most abundant phyla within the water microbiomes were Cyanobacteria, Proteobacteria, Actinobacteria, and Bacteroidetes, which comprised on average 55.4, 36.5, 5.6, and 0.7% of the microbial communities, respectively (Figure 1C). Members of Cyanobacteria such as dominant *Microcystis aeruginosa* were significantly more abundant at the water surface, while members of Proteobacteria, Actinobacteria, and Bacteroidetes were more abundant within the water column (*q* < 0.05 for each) (Figure 2A). In total, there were three microbial species (*M. aeruginosa*, *Microcystis viridis*, *Halomonas* sp. JS92-SW72) that were more prominent at the pond water surface and 36 microbial species (e.g., *Synechococcus* sp. CBW1004, *Limnohabitans* sp. 103DPR2*, Magnetospirillum gryphiswaldense, Microcystis* sp. MC19*, Phenylobacterium parvum, Acidovorax* sp. T1) that had higher relative abundances in the water column (*q* < 0.05; Supplementary Table S1).

The pond sediments harbored microbial communities with the highest α -diversity with comparable abundances of Proteobacteria to the water column microbiome, along with higher abundances of Actinobacteria and Firmicutes and lower abundances of Cyanobacteria, suggesting gradient ecological dynamics with microbial settling and resuspension (Figure 1C). There were 25 species that were significantly more abundant in the water column than the in the sediments, such as *M. aeruginosa* and *M. viridis*, which were further dominant at the water surface. Alternatively, there were 11 microbial species enriched in the sediment microbiome, including *Serratia marcescens*, *Rhizobacter* sp. AJA081-3, *Shinella* sp. XGS7, *Methylocystis parvus, Rubrivivax gelatinosus*, among others (*q* < 0.05; Supplementary Table S1).

**Figure 3:**
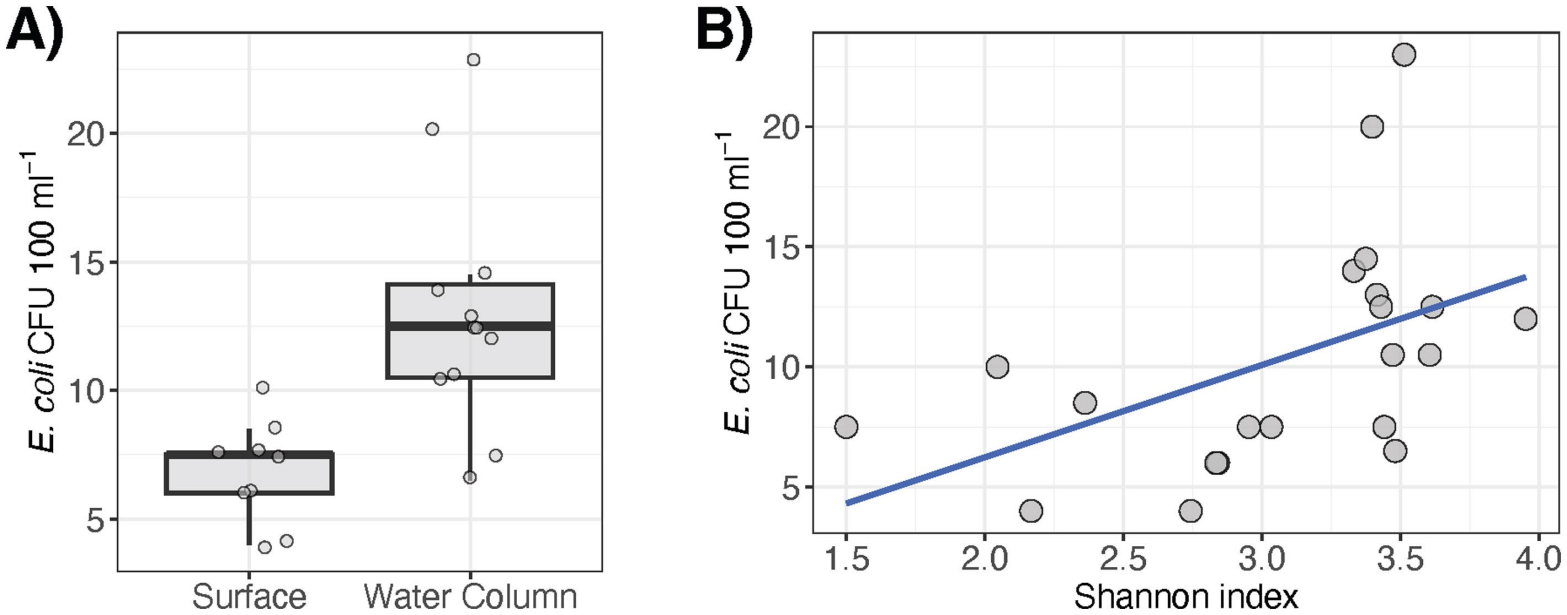
*E. coli* variation in pond water correlates with **(A)** sampling depth (*p* = 0.001) and **(B)** microbiome α-diversity (*rho* = 0.519, *p* = 0.016).

As described in our previous report (Stocker et al., 2024), the set of studied water quality properties also exhibited spatial variation throughout the pond. Here, most of these measured water variables correlated with taxonomic profile α -diversity (Table 1), which may be attributed to confounded differences in both water quality and microbial diversity at the surface and within the water column (Figure 1A). For example, water temperature was lower at increasing depths and inversely correlated with water microbiota α -diversity (*rho* = −0.686, *p* = 0.001), perhaps reflecting microbial resuspensions around the underlying sediments. In addition, *E. coli* (CFU 100 mL^−1^) was more abundant in the water column than at the surface (*p* = 0.001) and correlated with α -diversity (*rho* = 0.519, *p* = 0.016) (Figure 3). Moreover, water properties linked to proliferation of algae or Cyanobacteria (e.g., chlorophyll and phycocyanin contents, colored dissolved organic matter), which was dominant at the surface (Figure 1C), were significantly associated β -diversity of the water microbiomes as well (Table 1).

**Table 1:** Metagenome correlations with pond water quality properties.

Water property	Microbial taxa α-diversity		Microbial taxa profile		Functional (MetaCyc) profile	
	*rho*	*P*	*R* ^2^	*P*	*R* ^2^	*P*
Temp, °C	**−0.686**	**0.001**	0.063	0.240	0.064	0.254
DO	**−0.757**	**<0.001**	0.062	0.234	0.085	0.176
SPC	0.344	0.138	0.046	0.407	0.073	0.181
pH	**−0.760**	**<0.001**	0.056	0.330	0.048	0.362
NTU	**−0.589**	**0.007**	0.088	0.164	**0.192**	**0.015**
YSI-PC	**−0.749**	**<0.001**	**0.398**	**0.002**	**0.377**	**0.001**
YSI-CHL	**−0.698**	**0.001**	**0.413**	**0.001**	**0.368**	**0.001**
*f*DOM	**0.674**	**0.001**	**0.193**	**0.015**	**0.213**	**0.012**
PO4	0.205	0.371	0.054	0.293	0.026	0.693
NH_3_	**0.745**	**<0.001**	0.102	0.098	**0.145**	**0.031**
NO_3_	0.149	0.517	0.053	0.304	0.033	0.579
ECCFU	**0.519**	**0.016**	0.094	0.116	**0.149**	**0.030**
TC	0.243	0.287	0.030	0.627	0.028	0.628
TIC	**0.469**	**0.033**	0.046	0.382	0.060	0.259
TN	**0.608**	**0.004**	0.096	0.102	0.082	0.153
TOC	−0.042	0.858	0.023	0.765	0.031	0.622
CDOM	**−0.679**	**0.001**	**0.291**	**0.003**	**0.259**	**0.002**
INV	**−0.584**	**0.006**	**0.306**	**0.001**	**0.252**	**0.003**
PHYC	**−0.808**	**<0.001**	**0.288**	**0.002**	**0.248**	**0.005**

Significant associations (*p* < 0.05) are displayed in bold font.

### 3.2 Pond microbiome functional potential

In line with trends for the microbial taxa, the encoded metabolic profiles significantly differed between the surface and within the water column (PERMANOVA *R*^2^ = 0.196, *p* = 0.013; Supplementary Figure S2), though not at the different sampling times of day (PERMANOVA *R*^2^ = 0.101, *p* = 0.400). As water quality also differed across scale, particularly between the surface and deeper into the water column, the water metagenome functional profiles correlated with many of the measured variables (Table 1). In addition to significantly correlating with the same water properties as microbial taxonomic profiles, functional profiles further exhibited significant associations with NTU (PERMANOVA *R*^2^ = 0.192, *p* = 0.015), NH3 (PERMANOVA *R*^2^ = 0.145, *p* = 0.031), and ECCFU (PERMANOVA *R*^2^ = 0.149, *p* = 0.030), suggesting that microbiome functional diversity may be more tightly linked to water quality than microbial taxa composition and structure.

There were 41 and 40 total metabolic pathways that were enriched (*q* < 0.05) at the surface vs. within the water column (*q* < 0.05; Supplementary Table S2). At the pond surface, pathways involved in photosynthesis (e.g., photosynthesis light reactions, oxygenic photosynthesis, Calvin-Benson-Bassham cycle) and nucleotide biosynthesis (e.g., synthesis of pyrimidines, purines, adenosine and guanosine) were significantly more abundant (Figure 2B). Alternatively, pathways involved in respiration (e.g., pentose-phosphate pathway, pyruvate fermentation, glycolysis) and amino acid biosynthesis (e.g., isoleucine, valine, branched amino acids) were more abundant in metagenomes in the water column (Figure 2B). These trends extended into water column-sediment dynamics. There were 46 pathways significantly more abundant in the water column (e.g., pentose-phosphate pathway, oxygenic photosynthesis, Calvin-Benson-Bassham cycle, hydrogen production), while 27 were enriched in the underlying sediments (e.g., pyruvate fermentation, synthesis of pyrimidines, purines, adenosine and guanosine) (*q* < 0.05 for each; Supplementary Table S2).

**Figure 4:**
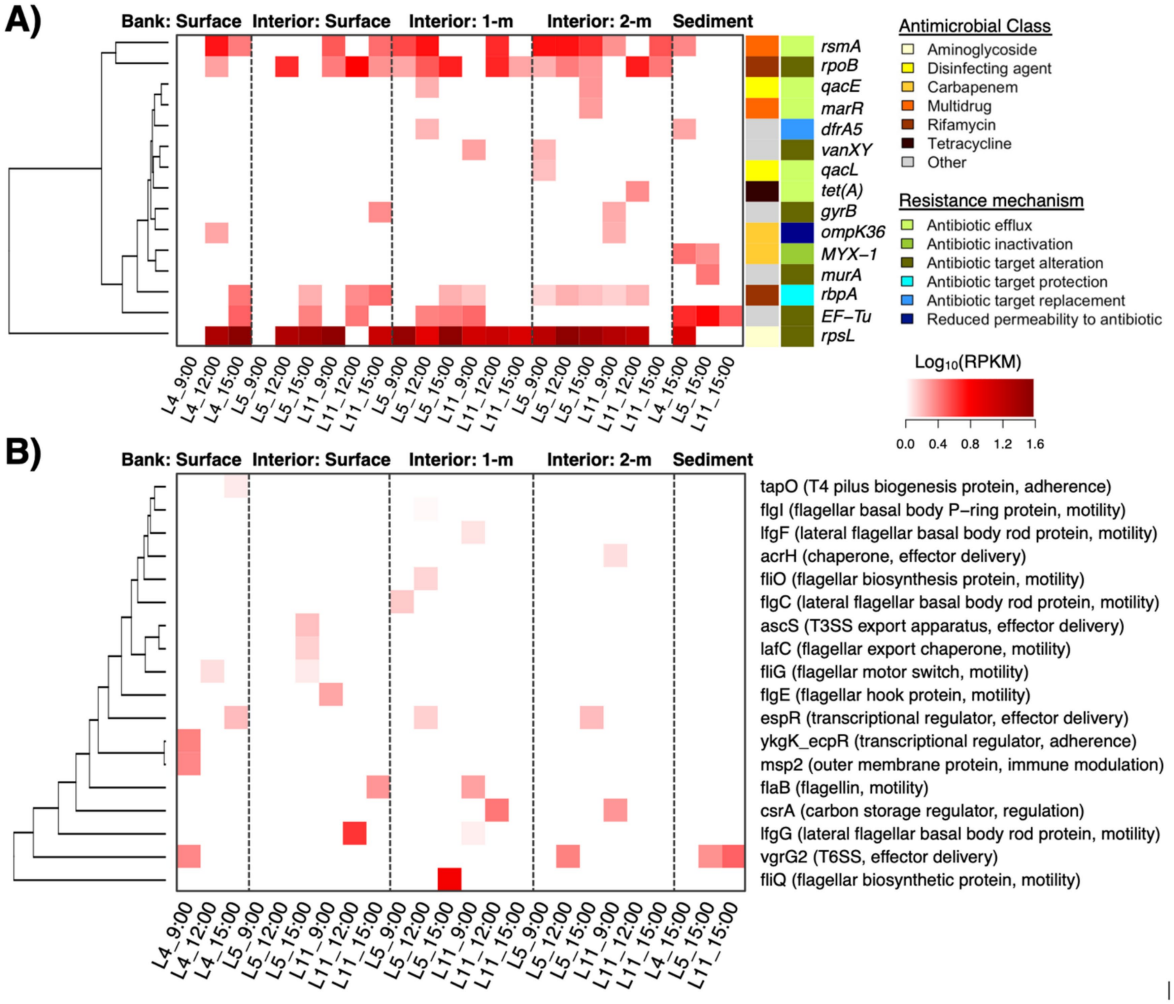
Antimicrobial resistance (AMR) genes (Panel **A**; row side colors indicate the antimicrobial class and mechanism associated with resistance) and virulence factors (Panel **B**; gene function in parenthesis) that were detected in the pond metagenomes. Heat color indicates relative abundance as log-transformed reads per kilobase per million reads (RPKMs).

We further identified 15 AMR and 18 VF genetic elements within the pond metagenomes. The AMR elements encoded resistance to aminoglycosides, carbapenems, rifamycin, tetracyclines, and multidrug efflux. Spatial distributions of certain genes were generally sporadic and there were several instances of detection in only one sample type (e.g., *marR, qacL,* or *tet*(*A*) at the 2 m water depth) (Figure 4A). Only four AMR genes were detected in water samples across all pond depths (i.e., *rsmA*, *rpoB*, *rbpA*, and *rpsL*) and most of these commonly occurring genes were largely housekeeping features that may be involved in AMR via antibiotic target alteration under specific mutations. Moreover, notable AMR genes that were detected in pond water and not sediments included *qacE* that is involved in resistance to disinfecting agents via efflux, *ompK36* that can result in reduced permeability to carbapenems, and *gyrB* and *vanXY*, both of which are conferred via target alteration. On the other hand, AMR genes detected in sediment samples and not the water column were with *MYX-1* and *murA*, which encode enzymatic inactivation (carbapenemase) and potential antibiotic target alteration, respectively. Moreover, the VFs primarily consisted of flagellar machinery for motility, effector systems (e.g., T3SS, T6SS), and proteins involved in adherence (Figure 4B). In contrast to the metabolic pathways that were differentially abundant at the surface and within the water column, the relative abundances of AMR and VF genes detected were low and did not correlate with the sampling factors or the water quality properties measured in this study (*q* > 0.05 for each genetic element).

### 3.3 MAGs reflect dominant taxa carrying AMR and virulence traits

**Figure 5:**
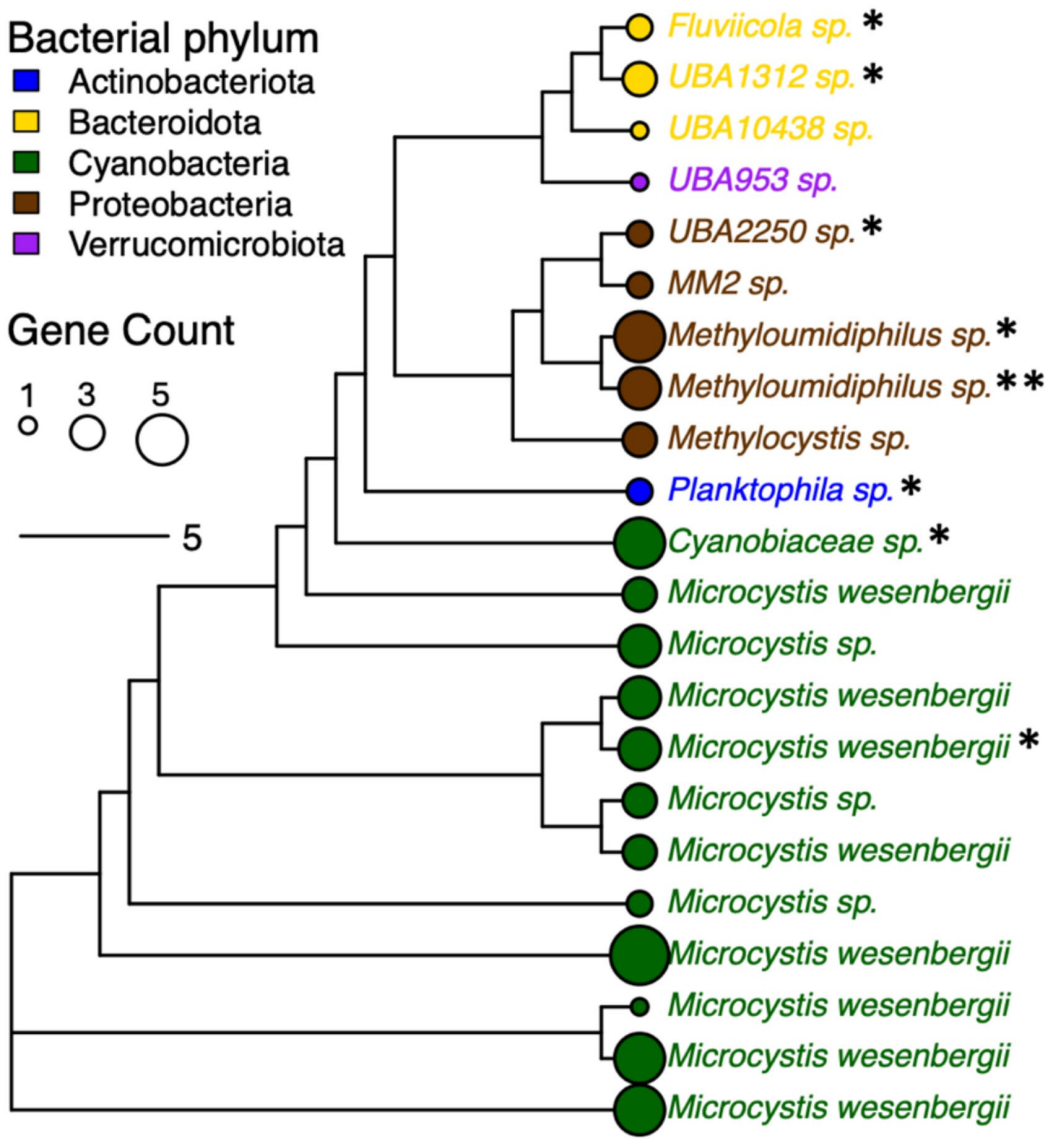
Phylogenetic tree for bacterial strains (distinguished by 99% ANI) recovered from pond water metagenomes based on core gene alignment. MAGs without species assignment are indicated with “sp.” and considered novel based on relative evolutionary distance (RED) score provided by taxonomic assignment with GTDB-Tk. Node size corresponds to numbers of genes encoding proteins with homology to AMR genes (indicated by total “*”), VFs, and additional stress response features. Node and font color indicates bacterial phylum.

Processing the metagenomes with *de novo* assembly yielded 67 MAGs with at least medium-quality (i.e., predicted completeness >50% and contamination <5%) that were recovered from the water microbiomes, with at least one MAG recovered from each sample. Dereplication at 99% ANI yielded 22 distinct strains spanning Actinobacteriota (*n* = 1; referred to as Actinobacteria in the built database for the Kraken2 analysis), Bacteroidota (*n* = 3; referred to as Bacteroidetes in the built database for the Kraken2 analysis), Cyanobacteria (*n* = 12), Proteobacteria (*n* = 5), and Verrucomicrobiota (*n* = 1) (Figure 5). These taxa comprised 12 non-redundant species, all of which were considered novel taxa based on ANI and RED values, i.e., the strain for *Microcystis wesenbergii* in Figure 5 clustered with *Microcycstis* sp. at the species-level (>95% ANI). The frequency of Cyanobacteria and Proteobacteria strains among the recovered MAG was consistent with their dominance among the pond microbiomes (i.e., 54.6 and 22.7% of MAGs and 55.4 and 36.5% in Kraken2/Bracken, respectively).

The various strains encoded several genes with homology to genes associated with AMR (*n* = 6), VFs (*n* = 2), and stress response (*n* = 18) (Supplementary Table S3). The AMR features identified among MAGs were involved in resistance to beta-lactams (*blaOXA, blaPAU*), tetracycline (*tetA(58)*), trimethoprim (*dfrA3*), pleuromutilin (*taeA*), or multidrug efflux (*ranA*). The VFs included *bpsD* and *katP*. The stress response features included biocide resistance via multidrug efflux (e.g., *ssmE, smr, qacE, qacF*), heat shock (*clpK*), and a variety of genes involved in tolerance to metals such as nickel and copper (Supplementary Table S3). Thus, there were consistencies in key features identified with the more general read-based characterization of metagenomes with ShortBRED described earlier, such as *dfrA5, acrH, tetA, qacE,* and two genes associated with resistance to beta-lactams or carbapenems (Figure 4A). At least one AMR genetic element was found to be associated with 66.7 and 60% of strains representing Bacteroidota (*n* = 2/3 MAGs) and Proteobacteria (*n* = 3/5 MAGs), while for Cyanobacteria this was only 16.7% of strains (*n* = 2/12 MAGs) (Figure 5). Although specific gene RPKMs were not correlated with sampling factors, perhaps reflecting sequencing depth limitations as described earlier, the observed trends in MAGs suggested that AMR may be disproportionately associated with the Proteobacteria and other water column/sediment-enriched taxa. Nevertheless, we were able to link AMR, virulence, and stress response genes to the range of dominant strains throughout the agricultural pond.

## 4 Discussion

The physicochemical properties of agricultural waters fluctuate spatiotemporally, concurrent with shifts in microbial community dynamics (Stocker et al., 2024). While the US FDA has implemented recommendations for farm-scale monitoring of pre-harvest water quality to enhance applications for food safety (U.S. Food and Drug Administration, 2024), understanding how specific sampling strategy and design may impact observations is important for practical risk management decision-making frameworks. The present study aimed to characterize metagenomes of agricultural pond water as a factor of sampling location, water depth, and time of day. Microbial α -diversity was lowest at the water surface, where members of Cyanobacteria were most abundant, and increased in the water column toward the sediments. In line with the surface-enriched phytoplankton, we observed correlations between microbiome β -diversity and phycocyanin, chlorophyll-a, and concentrations of *f*DOM and *c*DOM. Previous studies on lake microbiota have also demonstrated that DOM plays an important role in shaping microbial community composition (Crump et al., 2003; Kent et al., 2006; Amaral et al., 2016; Hengy et al., 2017). Moreover, shifts toward a more diverse microbiome within the water column, thereby being inversely proportional to DO, were consistent with previous reports for lakes (Bernard et al., 2019). Overall, the pond water microbiota correlated with water quality properties that differed between the pond surface and within the water column.

Similarly, the pond water microbiome functional potential was depth-dependent and correlated with many of the measured water quality variables as well. This was somewhat expected, as a stratification within water bodies gives rise to distinct changes in light, temperature, DO, and pH (i.e., epilimnion at the surface and hypolimnion with depth) (Fenchel and Finlay, 2008). Metabolic pathways at the surface prominently featured photosynthesis and nucleotide biosynthesis, while those in the water column and sediments transitioned to increases in respiration, the non-oxidative branch of the pentose phosphate pathway (PPP), and amino acid biosynthesis. Cyanobacteria and other dominant water microflora can shift metabolic pathways in response to predation and environmental cues, which may follow diurnal patterns (Pattanayak et al., 2020; Selim et al., 2021; Grzesiuk et al., 2022). When oxygen becomes limited, bacterioplankton can perform anaerobic glycolysis via the PPP to produce NADPH and other metabolic intermediates (Mckinley, 1977; Bjerkas and Bjornstad, 1999). Phytoplankton also activate PPP under dark conditions (e.g., in the water column with increasing depth) when Photosystem II cannot be utilized (Stal and Moezelaar, 2006). The role of microbiome metabolism in mediating water quality changes warrants further investigation.

Microbial diversity increased with pond water depth and was greatest in the underlying sediments, which are a sink for settling organic matter. Consistent with our observations, Firmicutes have been reported to often comprise a major fraction of lake and pond sediment microbiota (Wang et al., 2019; Merino et al., 2023). These taxa degrade cellulose and lignin of decomposing plant matter and are involved in denitrification (Zhang et al., 2019). Accordingly, levels of ammonia (NH₃) in the water column were higher than at the surface, which likely reflected flux from bacterial activity in the sediments (Leoni et al., 2018). As a sink for nutrients and microbiota diversity, sediments can also become a source due to diffusion and resuspension (Hassard et al., 2016; Dadi et al., 2017) that may play an important role in the water quality dynamics throughout the pond.

Developing an effective water quality monitoring strategy requires consideration of variation across potential sampling locations. While the proportions of bacteria at the water surface were relatively stable throughout the day (i.e., 9:00, 12:00, 15:00) at the pond bank (L4), larger shifts at the surface were noted for the interior sampling sites (L5 and L11). This may be related to *Microcystis* changing its vertical position in the water column via gas vacuoles in response to solar radiation, which was greater at surface-interior than surface-bank sites due to partial shading from vegetation at the perimeter of the pond (Smith et al., 2022). Limited water depths at the nearshore sites (e.g., approximately 0.3–0.5 m maximum) also inherently reduced the capacity for vertical translocation. Thus, microbial water quality toward the interior of agricultural ponds may have been more influenced by diurnal changes in light than that at the shoreline due to both broader depths and relatively limited cover. Future research at additional ponds or along more sampling time points is needed to better understand within-site spatiotemporal variation in microbiome interactions with water quality.

Water metagenome diversity correlated with turbidity and *E. coli* concentrations, both of which were greater in the water column than at the surface. As *E. coli* is a routinely monitored indicator organism, the moderate, yet significant positive association with microbiome α -diversity may have implications for water quality monitoring for food safety risk analysis. The emergence of AMR and virulence features among environmental microbiota is also an emerging concern for public health. A variety of associated genes were identified in the pond microbiomes. For example, variants in *rpsL*, which can confer resistance to streptomycin and other aminoglycosides via key mutations, were detected at the highest abundance and frequency in the pond. These results are consistent with the study by Chopyk et al. (2020) that described *rpsL* to be the most prevalent resistance element among 33 detected AMR genes across 9 different irrigation water sources in the United States. In addition, *rpoB* and *Ef-Tu* (i.e., genes also involved in resistance via potential mutations) were also widely detected in the water and sediments, respectively, which is consistent with previous reports on rivers, lakes, and subaqueous soils (Mittal et al., 2019; Zhao et al., 2019; Su et al., 2020). Whether these housekeeping genes that are implicated in potential AMR exhibited variants of clinical significance was outside of the scope of this work. While efflux pump *rsmA* was found in more than half of the samples collected in this work, which was also seen in other studies (Rout et al., 2024b), additional AMR genes associated with multidrug efflux and carbapenem or tetracycline resistance via enzymatic resistance mechanisms were detected sporadically in the pond and may have implications for food safety as well. Like most of the AMR features, the VFs identified (e.g., encoding adherence, flagellar motility, secretion system) were detected in a limited number of samples. Although the sources for these genetic elements remain unclear, emerging chemical contaminants (e.g., heavy metals, antibiotics and other antimicrobials) have gained attention for possible adverse impacts on environmental microbiomes, and even more in wastewater systems (Bueno et al., 2021; Guruge et al., 2021; Sharma et al., 2025). Exploring how chemical drivers may associate with AMR in agricultural resources, especially under changing environmental conditions, is needed to inform strategies to mitigate potential risks.

While differences in AMR genetic elements across sampling locations and depths were not elucidated in the scope of this work, perhaps reflective of limited sample size and sequencing depth, we were able to link key features to dominant microbial strains within Cyanobacteria, Proteobacteria, and other phyla. There were a variety of AMR, virulence, and/or general stress response genes found in all MAGs recovered, which is consistent with other reports (Huang et al., 2024; Jiang et al., 2024). Notably, AMR genes were less frequently carried by strains of the surface-associated Cyanobacteria compared to other phyla. The greater microbial diversity within the sediments and water column (e.g., where Proteobacteria and Bacteroidetes were more dominant) suggests these sampling locations as more of a hotspot for genes encoding AMR and other stress responses. As bottom sediments of streams, lakes, rivers, and wastewaters have been reported as key reservoirs for AMR genes and the mobile genetic elements that mediate their transmission (Calero-Cáceres et al., 2017; Hess et al., 2018; Chen et al., 2020; Eramo et al., 2020), similar dynamics may occur in agricultural water systems. Employing long read metagenomics in future studies may help to better understand whether mobile elements are co-localized with AMR or virulence genes, as well as key metabolic pathways (e.g., PPP), and potentially enriched among taxa that exhibit pond depth variation.

We recognize several limitations to this study. While we focused on discerning the influence of sampling factors on observed water metagenome diversity, future studies collecting samples from various agricultural water sources known to differ in intrinsic properties would be essential to better understand the relationship between water metagenomes and water quality. Moreover, although our metagenomic approach offered opportunity for non-targeted molecular surveillance, the sensitivity of AMR gene detection was limited, likely due to low abundances in the natural microbiomes. Deeper metagenomic sequencing is essential in future studies to determine whether AMR and virulence in agricultural waters may exhibit patterns across space and time. For example, a recent study that characterized broad diversity of 1,582 AMR genetic elements (representing 25 antibiotic classes) in 404 surface water samples in South America targeted >20 million metagenomic read pairs per sample (Huang et al., 2024), which was about two times greater than the reads obtained in this work. In addition, employing “quasi-metagenomics” that involves some extent of culture enrichments prior to sequencing (Commichaux et al., 2021; Kocurek et al., 2023) may be necessary to enhance strategies that will help provide the systems-level understanding between foodborne pathogens and water quality.

## 5 Conclusion

The microbiological and physicochemical quality of agricultural water has significant implications for food safety, including emerging concerns for transmission of genetic elements involved in AMR. However, correlations between specific biological and environmental parameters, which may concurrently exhibit spatial and temporal variation (e.g., changes with water depth and time of day), are not clear. We addressed this gap by incorporating metagenomics into a comprehensive water quality assessment of an agricultural pond. Microbial taxonomic profiles and metabolic pathways largely varied between the water surface and at one and two meter depths in the water column, perhaps reflecting microbial exchanges with the underlying sediments. A variety of genetic elements encoding AMR, virulence, and other forms of bacterial stress response were identified throughout the pond, some of which were linked to specific bacterial strains within the water microbiome. Overall, our findings highlight key differences in water metagenomes throughout the pond that significantly correlated with dynamic water properties (e.g., concentrations of chlorophyll, DOM, ammonia, culturable *E. coli*). Spatiotemporal variation in water quality must be considered when developing applications for molecular surveillance in food safety risk assessment frameworks.

## Data Availability

Raw sequencing data for shotgun metagenomes of pond water and sediment samples, along with the MAG genome assemblies, are available under NCBI BioProject accession number PRJNA1062331. Scripts and code that may be used to reproduce our analyses are available at https://github.com/rablaustein/2025_Wye_pond_metagenomes.

## Footnotes

^1^ http://huttenhower.sph.harvard.edu/kneaddata

^2^ https://github.com/vegandevs/vegan

## Supplementary material

Supplementary Figure S1 Map of sampling locations in Stocker et al. (2024). Water and sediment samples that were collected at locations 4, 5, and 11 (orange arrows) were processed for metagenomics analysis in the present study.

Supplementary Figure S2 Compositional dissimilarity or β-diversity of the relative abundances of MetaCyc pathways within water metagenomes. Colors represent sampling location/depth and shapes represent time of day.

Supplementary Table S1 Relative abundances of microbial species with an average abundance >0.1% across all samples at the water surface, in the water column, and in sediments of the agricultural pond. LME model statistics for differences between values in microbiomes at the water surface vs. in the water column and the latter vs. in sediments are displayed. Bold font indicates significance (*q* < 0.05).

Supplementary Table S2 Relative abundances of MetaCyc pathways encoded in the metagenomes at the water surface, in the water column, and in sediments of the agricultural pond. LME model statistics for differences between values in microbiomes at the water surface vs. in the water column and the latter vs. in sediments are displayed. Bold font indicates significance (*q* < 0.05).

Supplementary Table S3 MAG amino acid sequences with homology (>50% identity) to AMR, virulence, and stress response genetic elements.
