# Supplementary figures and images for: Water metagenomes reflect physicochemical water quality throughout a model agricultural pond
Source: Front Microbiol. 2025 Jun 4;16:1535096. doi: 10.3389/fmicb.2025.1535096 (PMC12174113; doi:10.3389/fmicb.2025.1535096)

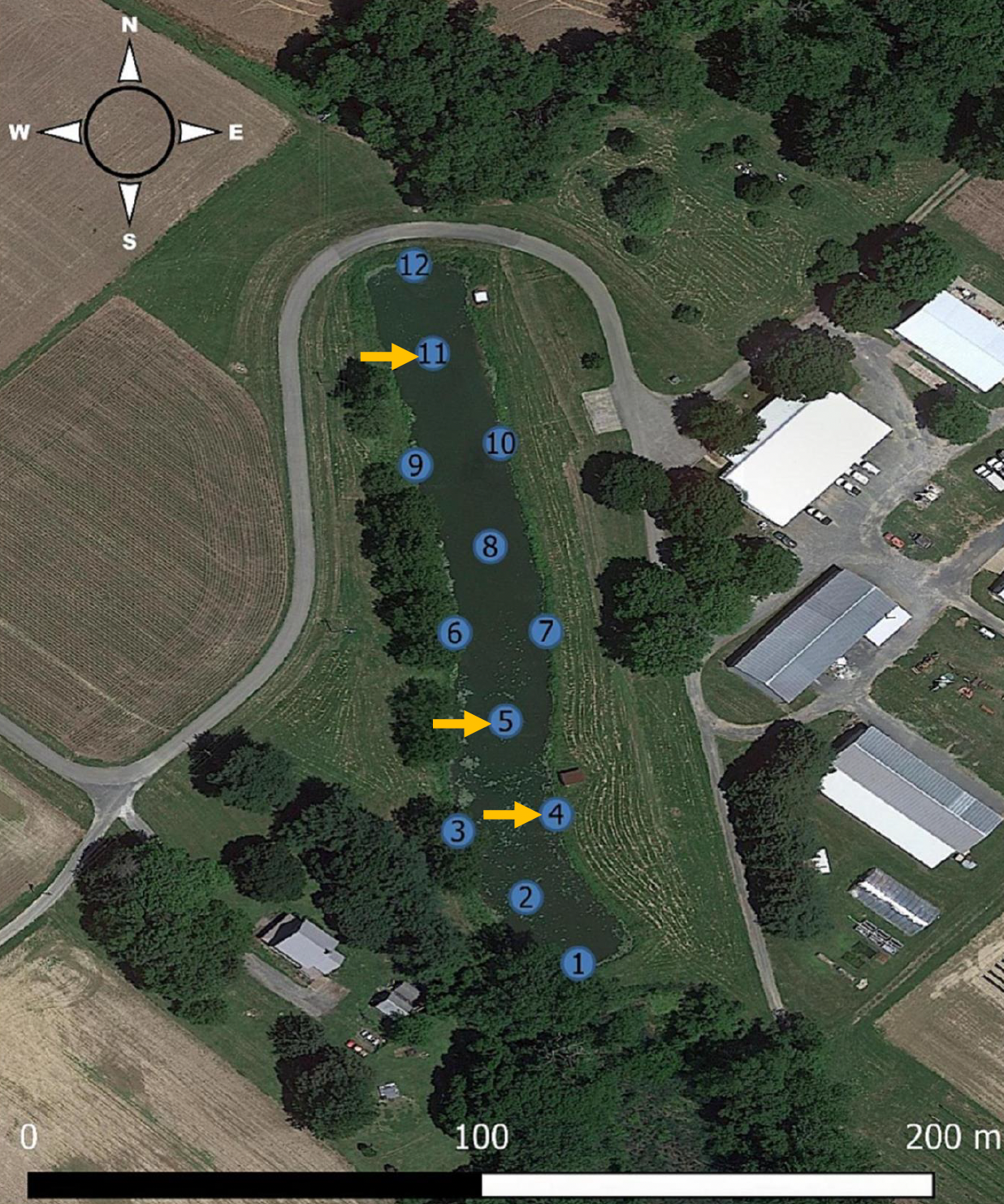

Supplement: Supplementary Figure S1 — Map of sampling locations in Stocker et al. (2024). Water and sediment samples that were collected at locations 4, 5, and 11 (orange arrows) were processed for metagenomics analysis in the present study. [file Supplementary_file_4.PNG]

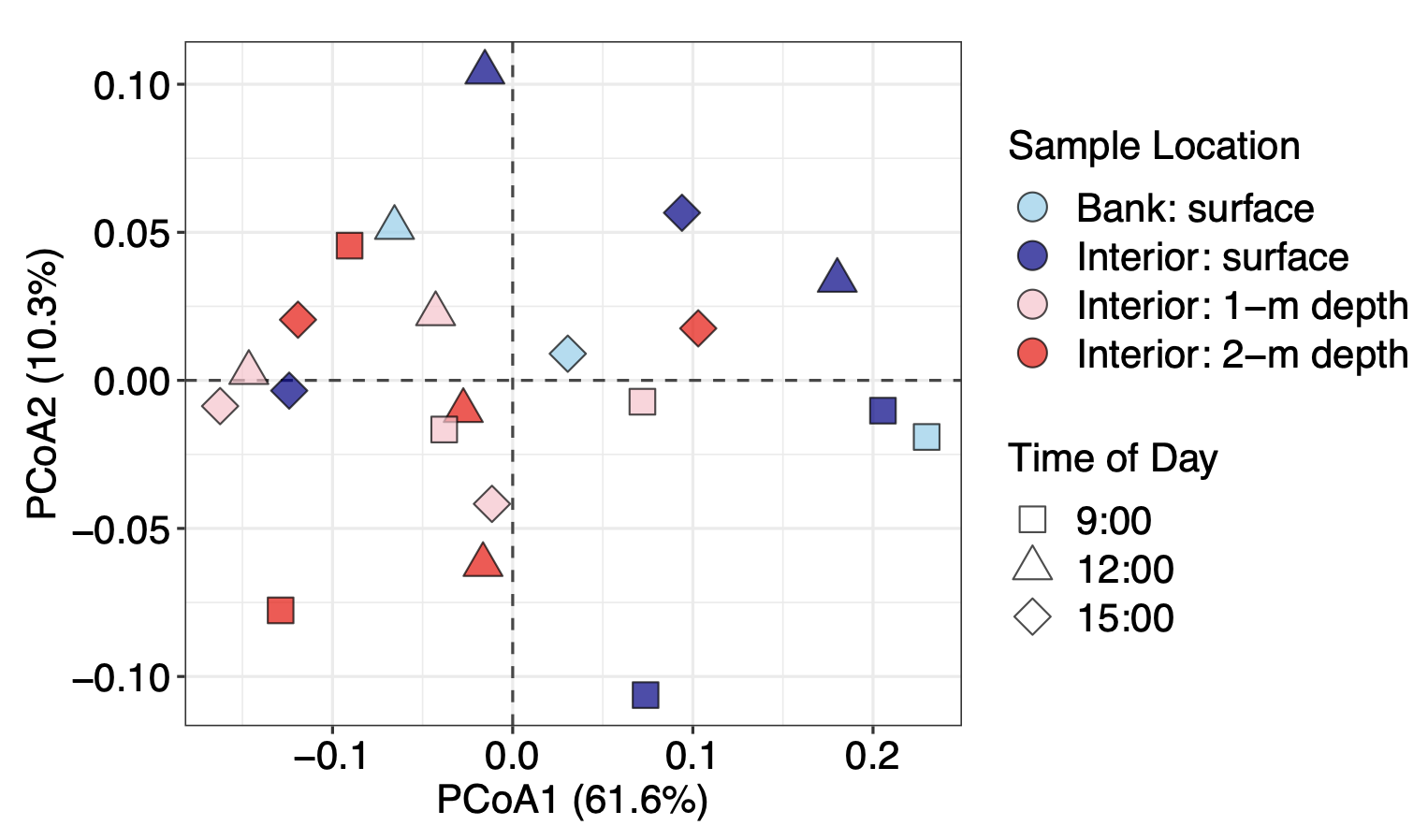

Supplement: Supplementary Figure S2 — Compositional dissimilarity or β-diversity of the relative abundances of MetaCyc pathways within water metagenomes. Colors represent sampling location/depth and shapes represent time of day. [file Supplementary_file_5.TIFF]
